# Lipids and Oxidative Stress Associated with Ethanol-Induced Neurological Damage

**DOI:** 10.1155/2016/1543809

**Published:** 2016-01-05

**Authors:** José A. Hernández, Rosa C. López-Sánchez, Adela Rendón-Ramírez

**Affiliations:** ^1^Tecnologico de Monterrey, Escuela Nacional de Medicina, 64710 Monterrey, NL, Mexico; ^2^Unidad de Biofísica (CSIC, UPV/EHU) and Departamento de Bioquímica, Universidad del País Vasco, Apartado 644, 48080 Bilbao, Spain

## Abstract

The excessive intake of alcohol is a serious public health problem, especially given the severe damage provoked by chronic or prenatal exposure to alcohol that affects many physiological processes, such as memory, motor function, and cognitive abilities. This damage is related to the ethanol oxidation in the brain. The metabolism of ethanol to acetaldehyde and then to acetate is associated with the production of reactive oxygen species that accentuate the oxidative state of cells. This metabolism of ethanol can induce the oxidation of the fatty acids in phospholipids, and the bioactive aldehydes produced are known to be associated with neurotoxicity and neurodegeneration. As such, here we will review the role of lipids in the neuronal damage induced by ethanol-related oxidative stress and the role that lipids play in the related compensatory or defense mechanisms.

## 1. Introduction

A clear relationship has been established between ethanol intake, addiction and dependency [1–3], and several risk factors for chronic disease and injury [4]. Indeed, the public health problem associated with increased alcohol consumption and alcoholism [5, 6] is becoming ever more severe due to the increased economic burden of the complications on the health national systems and the cost of the relevant treatments [4, 7–10]. Alcoholism provokes high rates of mortality and it increases in risk of several disabling disorders [4, 11]. Such damage can be classified in function of the organs involved (liver, kidney, heart, brain, etc.), the type of intake (acute or chronic), or the subject's age at the time of exposure to ethanol (prenatal, neonatal, or adult). In summary, ethanol has several negative health effects, especially if we consider prenatal exposure where the brain is a major target for the damage provoked.

## 2. Effects of Ethanol in the Brain

Ethanol has many effects in the brain depending on the age of exposure (prenatal, postnatal, or adult). For example, aggressive behavior and depression are observed after acute postnatal exposure to ethanol, possibly due to a decrease in circulating tryptophan, followed by the depletion of serotonin in the brain [12, 13]. Another effect of acute postnatal alcohol exposure is related to impaired impulsive and control behavior [14, 15], although few* in vivo* studies have focused on this issue. Cognitive performance has been associated with specific prefrontal cortical regions in Rhesus Macaque monkeys [11] and GABA receptors in this structure have been implicated in the effects of acute postnatal ethanol exposure [16–18]. Indeed, GABA was found to be a mediator in ethanol-induced ataxia [18, 19].

The most severe alcohol-related damage is found following acute prenatal or chronic pre- and postnatal ethanol exposure, effects that have been associated with a loss of neurons (Table 1). In terms of prenatal exposure, the babies born to women that drink alcohol excessively during pregnancy may suffer from fetal alcohol syndrome [20], a condition characterized by specific craniofacial abnormalities, pre- and postnatal growth deficiencies, and nervous system dysfunction that is manifested as persistent intellectual, behavioral, and neurological defects [5, 21]. These latter symptoms have been related to neurodegeneration in experimental animal models (see Table 1 for a summary of some of the available literature).

Chronic alcohol exposure has been associated with permanent neuronal loss in brain regions like the hippocampus and cerebellum. Moreover,* in vivo* studies have demonstrated neurological effects following chronic ethanol exposure in young and adult populations, with deterioration in memory, motor function, cognition, and so forth. All these effects could be due to neurotoxicity or neurodegeneration, and there is evidence that oxidative stress associated with ethanol metabolism is involved.

## 3. The Pharmacokinetics of Ethanol

The ethanol concentration that can be found in blood following its ingestion depends on its pharmacokinetics (PK). PK determines not only the time-course and persistence of ethanol in blood but also the amount of alcohol and its metabolic products that accumulate in different tissues, and hence their pharmacological and toxicological responses [48].

### 3.1. Absorption

In adults, the ethanol ingested is almost completely and instantly absorbed by passive diffusion, reaching a peak concentration in humans between 30 and 90 min. Absorption is more efficient in the small intestine than in stomach [49], a difference in absorption that is due to two factors. First, the thickness of mucus that protects the stomach appears to have a resistance ~16 times greater than that which protects the small intestine [49, 50], which also has a greater intestinal absorption surface due to the presence villi and microvilli [51]. The second difference reflects the speed of stirring caused by peristalsis, which is more important in the small intestine than in the stomach, playing a role in gastric emptying and in the intestinal transit time [49].

In addition, the presence of food is another factor that modifies the absorption rate [52], mainly as food reduces gastric emptying and ethanol is absorbed more slowly [53]. Solid food intake can reduce the ethanol absorption rate by 30% and it has been suggested that this effect is due to the need for food digestion prior to absorption process. As such, if food is taken in as a liquid then it would not produce this effect [49, 54]. Moreover, a small amount of ethanol can be oxidized to acetaldehyde by alcohol dehydrogenase (ADH) classes I and IV [52, 55] in the stomach and intestine. This acetaldehyde can be absorbed along with ethanol and metabolized by the liver or other tissues.

### 3.2. First Pass Metabolism and the Distribution of Ethanol

The amount of alcohol in any given tissue depends on its relative concentration in the blood, which is a function of first pass metabolism [49], that is, the oxidation of ethanol in the stomach, intestine, and liver.

Most first pass metabolism occurs in the liver [49, 55] and the rate-limiting step is the oxidation of ethanol to acetaldehyde. This reaction is catalyzed by proteins of the ADH family [56], of which class I (ADH1) and III (ADH3) enzymes metabolize ethanol in the liver [57, 58]. These two types of enzymes differ in their Km, with ADH1 having a low Km while ADH3 has a high Km value [57, 59]. Consequently ADH3 plays a more important role in the metabolism of alcohol at high concentrations. In addition, microsomal ethanol oxidizing system (MEOS) and catalase contribute to the metabolism of alcohol in specific circumstances, such as high ethanol concentrations [48, 60].

**Figure 1:**
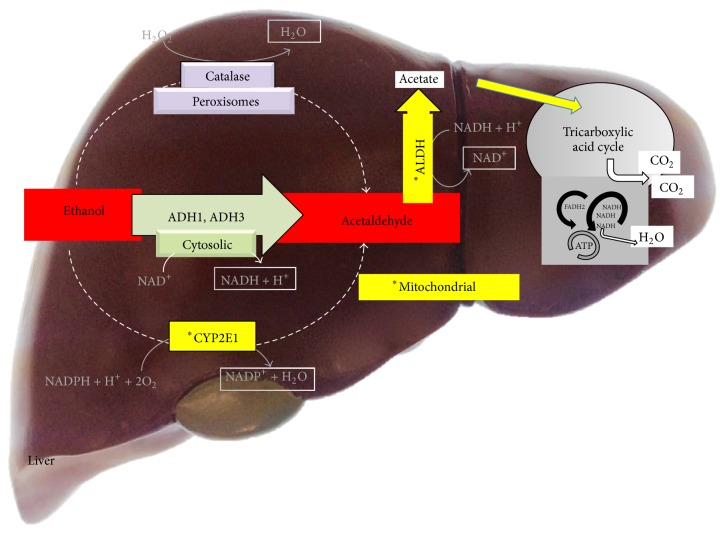
Mechanisms of ethanol metabolism in the liver. Alcohol dehydrogenase (ADH) and aldehyde dehydrogenase (ALDH) are the main enzymes that convert ethanol to acetate in the liver.

The acetaldehyde produced by the oxidation of ethanol is thereafter transformed to acetate by aldehyde dehydrogenase (ALDH) [61], which can be further metabolized through the tricarboxylic acid cycle to generate energy, or these metabolites can be deposited in the plasma [62, 63]. Indeed, increases in acetate but not acetaldehyde can be detected in human plasma after ethanol intake [64, 65] (Figure 1).

The efficiency of ethanol metabolism is dependent on the enzymatic activity and pathways involved. It has been reported that ADH, cytochrome P450 (CYP), and ALDH show genetic variations (ADH1B, ALDH2, CYP2E1^*∗*^6, and CYP2E1^*∗*^7B besides others) that affect enzymatic activity in the liver and alcohol metabolism [66–68]. As a result, ethanol's pharmacokinetic and pharmacodynamic properties are affected by this genetic variation, as reflected in interracial and ethnic pharmacological differences [56, 66–71]. Consequently the risk of developing diseases may increase in certain populations, including that of hypertension [70], alcohol dependence, and several types of alcohol-related cancer [60, 72–75].

After first pass metabolism, the remaining ethanol and its metabolites are distributed in different tissue, and the excess alcohol is excreted in the breath, urine, and sweat [56]. The distribution of ethanol throughout the body is driven in direct proportion to water content of each tissue, especially at the ethanol steady-state. Since ethanol is a small, polar molecule, the distribution volume of ethanol is dependent on the total body water of an individual (50 to 60% lean body weight) [76–78]. The variation in the distribution volume of ethanol has been evaluated for women and men, and in both sexes, the distribution volume decreases as the body mass index increases [79].

Alcohol-driven physiological changes, such as vascular effects (vasodilation) or changes in cardiac output, can also modify tissue blood flow and ethanol distribution [78]. Since the blood flow to the brain remains relatively constant, changes in the blood concentration of ethanol are the most relevant factor influencing the amount of ethanol delivered to the brain and therefore for the different levels of brain intoxication [78–80].

The distribution of ethanol is also particularly relevant during pregnancy, as 1-2  hours after maternal alcohol ingestion the fetal alcohol concentrations reach levels that are nearly equivalent to the maternal levels [81]. The elimination of ethanol by the fetus is impaired due to its reduced metabolic capacity. Thus, fetal exposure is prolonged through the reuptake of amniotic-fluid containing ethanol [81]. Ultimately, the elimination of alcohol from the fetus relies on the mother's metabolic capacity, which inevitably is a process that occurs late, meaning that the fetus is exposed to the toxicological effects of alcohol [82]. Therefore, many of the physical effects of ethanol on brain structure not only affect neurobehavioral features during fetal development but may also persist into childhood, potentially enduring until adulthood [82, 83].

### 3.3. Ethanol and Acetate Can Reach the Brain

Ethanol can cross the blood-brain barrier and it can be metabolized in the brain. Indeed, ethanol has been found in the human brain after alcohol intake [84], although metabolites of ethanol, like acetate, can also reach the brain as products of first pass metabolism [85]. Recently, the metabolism of [2-(13)C]-ethanol was evaluated in the brains of rats, and products such as labeled acetate, glutamate, glutamine, and GABA were detected found [86].

## 4. Metabolism of Ethanol and Acetate in the Brain

The oxidation of ethanol to acetaldehyde can occur in the brain through pathways that involve catalase, cytochrome CYP2E1, and ADH. The main pathway to metabolize ethanol in the liver is that involving ADH, although it has not been definitively shown to play a role in ethanol metabolism in the brain. In certain regions of the adult rat, mouse, and human brain it has been possible to identify ADH mRNA transcripts, with ADH1 and ADH4 expressed at distinct sites [87, 88], yet with no detectable activity after exposure to ethanol. Nonetheless, ADH4 inhibition avoids the synaptic dysfunction associated with severe alcohol intoxication in the hippocampus [89]. Moreover ADH activity (ADH1, ADH3, and ADH4) was found in the human brain but under pathological process like brain cancer [73] and Alzheimer's disease [90], and not induced by alcohol intake. In addition, and despite fulfilling a less prominent role in ethanol metabolism [85, 91], ADHs have been related to enhanced voluntary alcohol intake in rats [92].

Other pathways metabolize ethanol in the brain. Catalase and CYP2E1 are the main pathways; there is evidence that they do indeed play an important role in ethanol oxidation to acetaldehyde in the brain [91]. Indeed, acetaldehyde production in the brain* in vivo* depends on catalase activity [85, 93] and catalase appears to be expressed in all neural cells. Peroxisomal catalase is a tetrameric, heme-containing enzyme that, in addition to converting hydrogen peroxide (H_2_O_2_) to water and oxygen, can also oxidize ethanol to acetaldehyde. The discovery of the catalase pathway for acetaldehyde formation in the brain represented an important first step in our understanding of the role of acetaldehyde in the effects of ethanol in the brain [94]. Studies using inhibitors of catalase and acatalasemic mice revealed that catalase is responsible for approximately half of the ethanol metabolism occurring in the CNS [91]. Indeed, inhibitors of catalase are also effective in inhibiting the production of acetaldehyde.

The cytochrome P450 enzymes (CYP2E1) that are involved in ethanol metabolism in the liver have also been implicated in its metabolism in the brain. CYP2E1 reduces molecular oxygen to water and thus ethanol is oxidized to acetaldehyde. This enzyme is induced in response to chronic drinking and it may contribute to the increased rates of ethanol elimination in heavy drinkers. Some endogenous substrates for CYP2E1 include acetone and fatty acids, both of which are abundant in the brain [95]. The CYP2E1 system fulfills an important role in the generation of reactive oxygen species (ROS) and exposure to ethanol is related to the accumulation of ROS, which in rat brain homogenates may be attributed to the induction of CYP2E1 [96]. Not only ethanol but many other substrates are also metabolized by CYP2E1, including neurotoxins or procarcinogens, producing reactive intermediates [97, 98]. Moreover, in human neurons CYP2E1 is known to generate ROS and nitric oxide through the induction of NADPH/xanthine oxidase and nitric oxide synthase [99].

Therefore, CYP2E1 and catalase are the main pathways in the brain that metabolize ethanol to acetaldehyde, while ADH appears to play a minor role. Acetaldehyde is a biologically active compound and it has been implicated in alcohol addiction [100, 101], as well as inducing euphoria at low concentrations [102]. The effects of ethanol are modulated by acetaldehyde [100, 103], which in turn may react with endogenous substances to form other biologically active compounds. Acetaldehydes along with other proteins (adducts) were found in mice brain after alcohol consumption and in alcoholic human brains, suggesting they are involved in neural damage [104, 105]. Moreover adducts like salsolinol (formed when acetaldehyde binds to dopamine) were also seen to be involved in neurotoxicity [106] and in reinforcing addictive ethanol conduct [107]. Salsolinol has been identified in the brain and cerebrospinal fluid of patients with Parkinson disease, and it has been proposed to increase ROS production along with a reduction of glutathione [108], as well as reducing intracellular ATP and thereby acting as an inhibitor of mitochondrial energy supply. Thus, acetaldehyde reinforces its own effects or enhances the addictive action of ethanol [109, 110].

**Figure 2:**
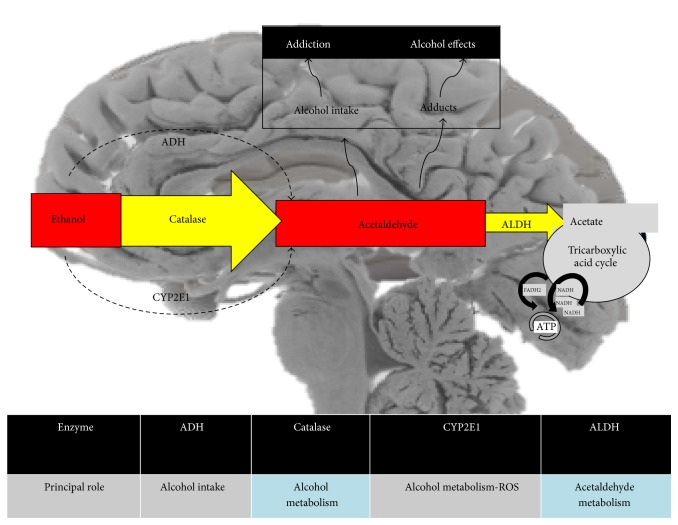
Enzymes related to ethanol metabolism in the brain and their principal role. Note the importance of acetaldehyde in ethanol metabolism.

As a result, acetaldehyde oxidation is required for detoxification and it can be metabolized to acetate by ALDH [111]. ALDH is critically important and the risk of alcohol-induced toxicity in individuals with mutant ALDH2 increases remarkably [112], while ALDH2 overexpression diminishes alcohol-related ROS production [113]. However, the accumulation of NADH increases in association with ALDH activity [114] and if the NAD^+^/NADH ratio decreases, the amounts of superoxide radicals increase [115, 116]. Moreover, although ALDH activity has beneficial effects, such as in the reduction of acetaldehyde, it also produces free radicals. Finally, the acetate produced by ALDH is metabolized in the Krebs cycle to produce energy or provide intermediaries for other molecules. Recent research showed that oxidation of [13]C-acetate generates specific neurotransmitters, as [13]C-glutamine, glutamate, and GABA levels were higher in chronic ethanol-exposed rats than in controls [86]. The production of these molecules may be related to the known effects of GABA receptors [16, 17, 19, 117], although other receptors are also involved in the effects of ethanol, such as dopamine, acetylcholine, and NMDA receptors [118–120] (Figure 2).

## 5. Oxidative Stress Produced by Ethanol

ROS are produced by exposure to ethanol [85] and they are associated with the effects of ethanol in the brain [92, 99, 101, 121–125], where ROS-related damage is due to oxidative stress [99, 124, 126–128]. The oxidative balance is a result of the amount of ROS that accumulates and the activity of antioxidant enzymes. In the brain, antioxidant enzymes are present in the cortex, cerebellum, hypothalamus, striatum, and spinal cord, and they include glutathione peroxidase, superoxide dismutase, glutathione reductase, and peroxiredoxin [129]. When the oxidative balance is disturbed, oxidative stress develops that affects the cell as a whole, as well as proteins, lipids, and DNA individually, provoking neurotoxicity or neurodegeneration.

## 6. The Antioxidant System and the Effects of Ethanol 

The formation of ROS accompanies many physiological processes, such that the body has developed a system of antioxidant protection against their harmful effects. In the brain, where the generation of free radicals is particularly severe, it is essential that the antioxidant system functions correctly [130]. Antioxidant activity is considered as enzymatic or nonenzymatic based on the mechanism of action involved.

### 6.1. Superoxide Dismutase (SOD)

It is an enzyme that catalyzes the dismutation of the superoxide anion to hydrogen peroxide, which is then decomposed by catalases primarily located in the peroxisomes. There are two main SOD isoenzymes found in the CNS of mammals: Mn-SOD (dependent on mitochondrial manganese ions) and Cu, Zn-SOD (SOD-1) present in the cytoplasm, microsomes, and synaptosomes [131]. Increased SOD activity is considered to be an adaptive response to oxidative stress, such as that induced by acute ethanol toxicity in the cerebral cortex [132]. However, acute ethanol intoxication reduces the activity of Cu, Zn-SOD in the cytosolic and microsomal fraction of the rat brain, and Mn-SOD activity in the mitochondria [131]. SOD interacts closely with catalase, which catalyzes the deprotonation of peroxide hydrogen and the oxidation of substances like methanol, ethanol, formate, nitrite, and quinones.

### 6.2. Catalase

In mammals, catalase is primarily located in the liver, erythrocytes, kidneys, and CNS. In the CNS, it can be found in microsomes [133] and it has been shown that, in acute ethanol poisoning, there is an increase of catalase activity in the cytosol, microsomes, and synaptosomes, as well as a reduction in the mitochondria of the rat CNS [131]. The increase in catalase activity following ethanol intake and its effects in the CNS are associated with weak ADH activity. This increase in catalase activity in the CNS may be adaptive processes induced by the increase in the hydrogen peroxide generated, as what occurs in the CNS of animals exposed to high concentrations of ethanol [134].

### 6.3. Glutathione Peroxidase (GSH-Px)

It is present in many tissues, as well as in the neurons and glia of the CNS [135, 136]. The role of GSH-Px is limited to the reduction of peroxides in which glutathione participates, which is accompanied by the formation of glutathione disulfide. In the rat and human CNS, the greatest glutathione peroxidase activity is observed in the gray and white matter of the cerebral cortex [137, 138].

### 6.4. Glutathione Reductase (GRed)

It is an enzyme present in the cytosol and in the mitochondria of most cells, catalyzing the regeneration of reduced glutathione oxidation at the expense of NADPH. Most GRed activity is found in neurons and glial cells [139], and acute ethanol poisoning significantly dampens GRed activity in the cerebral cortex [140].

The activity of antioxidant enzymes is significantly altered in the CNS of animals chronically intoxicated with ethanol. The antioxidative capacity of the CNS also depends on exogenous antioxidants obtained by the organism through its dietary intake. The most important exogenous antioxidant in the CNS is vitamin E, and both vitamin E and vitamin C content in the CNS falls after ethanol consumption, whereas vitamin A content increases [131].

## 7. Oxidized Fatty Acids as a Consequence of Oxidative Stress

Lipid peroxidation affects polyunsaturated fatty acids in membrane phospholipids as oxidative stress increases, producing bioactive aldehydes like 4-hydroxyalkenals and malondialdehyde [141]. Oxidative stress and the products of lipid peroxidation, 4-hydroxynonenal (HNE) [99, 142–145] or malondialdehyde [141, 146, 147], have been related to decreased neuronal viability in some studies. Ethanol-induced lipoperoxidation by oxidative stress [142] and its products decrease the intracellular reduced glutathione and increase its oxidized form [148]. HNE has also been associated with increases in mitochondrial permeability and cytochrome c release [143, 149, 150], the latter triggering apoptotic cell death by activating caspases [145, 150]. Interestingly, the toxicity mediated by the product of lipoperoxidation was weaker when glutathione transferase A4-4 activity was enhanced and glutathionyl-HNE was produced, avoiding the accumulation of HNE [150, 151] and possibly serving as a mechanism of tolerance. However, the activation of glutathione transferase A4-4 was suppressed in the presence of anionic phospholipids like cardiolipin [152]. Furthermore, the ability of HNE to produce glutathionyl-HNE was prevented by a PLA2 inhibitor [153], suggesting a role of PLA2 in the production of HNE.

## 8. The Role of Phospholipids in Stress Damage 

Cardiolipin is a phospholipid and it is the major component of mitochondrial membranes, although ethanol-induced oxidative stress provokes a loss of this lipid [152, 154–157] in conjunction with the appearance of HNE [157, 158]. Therefore, cardiolipin oxidation occurs following ethanol ingestion and consequently its fatty acids are released from phospholipids by PLA2. When cardiolipin is affected by ethanol, mitochondrial function is impaired and the outer mitochondrial membrane may disintegrate [157, 159], which could induce the release of cytochrome c from the mitochondria and trigger an apoptotic cascade mediated by caspases [158, 160]. Interestingly, the neurodegeneration induced by ethanol can be prevented by an inhibitor of PLA2* in vitro* [153, 161].

Phosphatidylserine (PS) has also been shown to play a role in apoptotic signaling, and both the reduction in PS and the enhanced neuronal cell death that ensues during the developmental period may contribute to the brain defects often observed in fetal alcohol syndrome [162]. Meanwhile, docosahexaenoic acid (DHA: 22:6n-3) prevents neuronal apoptosis by promoting PS accumulation [162], while conversely, PLA2 activity and oxidation-mediated HNE production may diminish the levels of PS.

## 9. Ceramide Related to Neurodegeneration

Ceramides are produced in the central nervous system by* de novo* synthesis or sphingomyelin hydrolysis [163]. Ceramide has been shown to accumulate in mitochondria upon the induction of apoptotic processes related to neurodegeneration [164–175]. The expression of serine palmitoyltransferase was localized in neurons and it was enhanced in caspase 3-positive neurons induced by ethanol [172], indicating that* de novo* ceramide synthesis participates in ethanol-induced apoptotic neurodegeneration in the brain. Although ceramide synthase 6 (CerS6) fulfills a protective role, this enzyme produces C16-ceramides and they are the precursors of other sphingolipids, such as sphingomyelin and glucosylceramide. Interestingly, CerS6 is enhanced within hours of ethanol withdrawal as a compensatory effect [176]. In summary, ceramide is an apoptotic signal [173] but it is also necessary for the sphingomyelin synthesis required to produce diacylglycerol (DAG), which in turn activates PKC [177], thereby avoiding apoptosis [178].

## 10. Lipids Potentially Involved in the Compensatory Mechanisms Protecting against Ethanol-Induced Damage

While some lipids are altered to signal cells for destruction, others seem to offset some of the effects that occur due to oxidation. For example, there is more cholesterol in neuron membranes exposed to ethanol [155]. Cholesterol is known to provide rigidity to membranes and ethanol is effective in disrupting unstable lipid membranes. Hence, an increase in the cholesterol present in membranes may represent a compensatory mechanism to combat ethanol damage. Indeed, when mitochondrial cardiolipin is oxidized and its fatty acid released, membranes become unstable due to a loss of rigidity.

Other lipids can also reduce the availability or the effects of metabolites of ethanol, such as phosphatidylethanolamine, phosphatidylethanol, and acylethanolamine. Ethanol exposure augments the amount of phosphatidylethanolamine due to the attachment of aminated ethanol to citidyldiphosphate [152, 179], resulting in the production of phosphatidylethanolamine through the Kennedy pathway [180]. Moreover, phosphatidylethanolamine can serve as a substrate for acyltransferases and indeed N-acylphosphoethanolamine (NAPE) is produced following ethanol exposure [168]. The amount of NAPE in membranes augments under cellular stress and as a result of tissue damage [181–184], and NAPE represents a precursor of the N-acylethanolamines [185] involved in learning and memory [186], neuroinflammation [187], oxidative stress, neuroprotection, and neurogenesis. Palmitoylethanolamine treatment of cultured cells produces neuroprotection against oxidative stress, impeding apoptosis [187–189] and protection in mice with chronic constriction injury [190]. Moreover, the endocannabinoid anandamide is also involved in neurodegeneration and thus acylethanolamines, and especially palmitoylethanolamine, appear to play an important role as neuroprotectors. Acylethanolamines can be found in the mitochondria* in vitro* [191] and palmitoyl requires carnitine to enter mitochondria. When cells or animals receive carnitine it acts as a neuroprotective agent, preventing ethanol-induced damage [147]. Furthermore, *ϖ* type-3 unsaturated fatty acids and DHA provide neuroprotection in conjunction with an increase in the formation of acylethanolamine [161, 162], suggesting that the formation of the latter prevents the damage caused by the oxidative metabolism of ethanol. Finally, ethanol can also be metabolized as phosphatidylethanol, a molecule found in the brain of rats [192] that is possibly formed to avoid ethanol oxidation.

## 11. Conclusions

**Figure 3:**
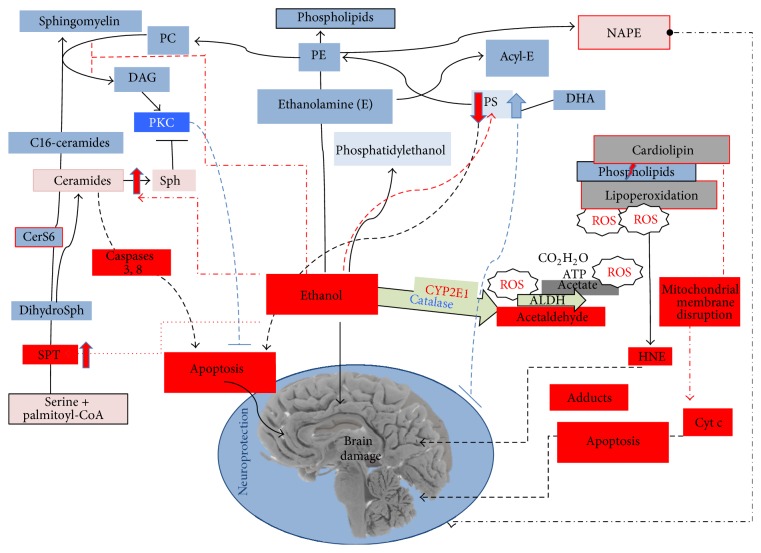
The role of lipids in ethanol-induced damage. Lipid metabolic pathways may be involved in neurodegeneration, such as lipoperoxidation, reduced phosphatidylserine (PS), N-acyl-PE (NAPE), and ceramide/Sph (sphingosine). Some lipids are produced as a compensatory mechanism and they fulfill a protective role, such as c16-ceramide, PS, sphingomyelin (SM), phosphatidyl ethanolamine (PE), and phosphatidylethanol.

**Figure 4:**
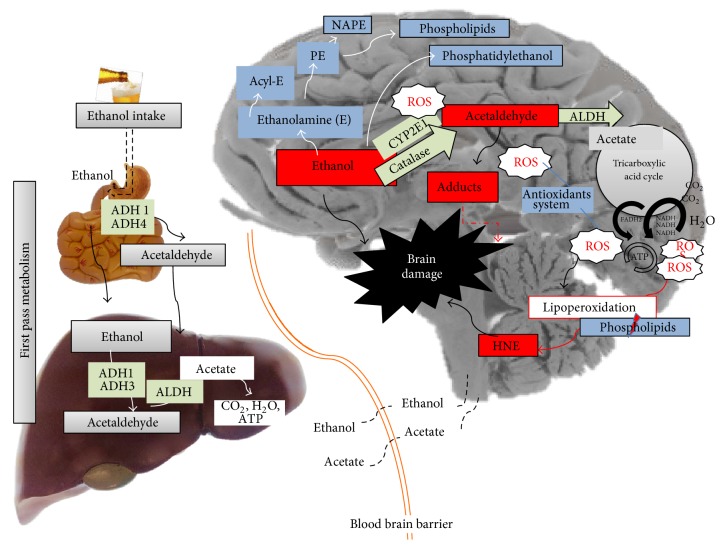
Oxidative stress and the role of lipids related to ethanol metabolism in the brain. Ethanol intake undergoes first pass metabolism in the stomach, intestine, and liver, although excess ethanol reaches the brain. Ethanol metabolism increases oxidative stress and lipid oxidation occurs, affecting mitochondrial membrane phospholipids and provoking cell death, thereby provoking damage in the brain. However ethanol-induced damage can be avoided by the activation of compensatory mechanisms involving lipids: E (ethanolamine), PE (phosphatidylethanolamine), acyl-E (acyl-ethanolamine), and NAPE (N-acyl-phosphatidylethanolamine).

Lipid metabolism is clearly affected by exposure to ethanol (Figure 3), and the alterations to lipid components like cardiolipin and some phospholipids in response to ethanol provide evidence of cell damage. The formation of oxidized species, abnormal lipids, and dysfunctional membranes due to ethanol uptake also provokes cell degeneration. However, compensatory mechanisms exist to dampen the effects of these metabolic events and to minimize cell damage, as reflected by the neuroprotective activities of natural lipids like DHA, esters, vitamin E, and so forth. Thus, ethanol-induced neurodegeneration is at least partly the result of the equilibrium maintained between the toxicity of signaling lipids and the protection they confer on the cell (Figure 4).

## Figures and Tables

**Table 1:** Summary of the neurological effects induced by *in vivo* ethanol administration at different ages.

Age of exposure	Species (model)	Frequency of administration	Structural CNS changes	Behavioral, intellectual, or other effects	References
Prenatal	Rat	Chronic	Reduced number of neurons and dendritic spines in the hippocampus and pyramidal tracts	Memory, spatial learning	[22]
			The cerebellum is most sensitive to alcohol-induced Purkinje cell loss	Cerebellar disorders (ataxia, cognitive, behavioral, and affective disturbances)	[23, 24]
			Cholinergic neurons loss	Spontaneous alternation, spatial working memory	[25, 26]
	Human	Chronic	Reduction in gray and white matter in the hippocampus, amygdala, thalamus, caudate, putamen, and globus pallidus	Cognitive, behavioral, and neurological impairments	[27, 28]

Young	Human	Chronic	Reduced white matter, corpus callosum, and hippocampal volumes	Impairment in neurocognitive tests, including those measuring memory, attention, visuospatial skills, and executive function	[29, 30]
			Reduced oxygen consumption in the subcallosal, anterior cingulate, left prefrontal, and bilateral insular regions	Dysfunction during spatial working memory and simple motor tasks	[31, 32]

Adult	Human	Chronic	Reduced volume in the diencephalon, cerebral cortex, hippocampus, and white matter	Progressive cognitive dysfunction and loss of neural plasticity due to reduced GABAergic inhibition and increased glutamatergic excitation	[33]
	Rat	Chronic	Corpus callosum ultrastructure	Cognitive and motor function	[34]
	Human	Chronic	Frontal and temporal lobes	Attention, impulsivity, verbal memory, and impaired cognition	[35, 36]
	Human or monkey	Chronic	Hypothalamus D3 and 5HT_1A_ neuronal receptors	Alcohol dependency	[37, 38]
	Human	Chronic	Nucleus basalis Meynert	Loss cognitive disorders and dementia	[39–42]
			Cerebellar atrophy, Purkinje cell loss	Wernicke's encephalopathy, cognitive and emotional dysfunction	[43, 44]
			Peripheral nerves stimulation	Withdrawal-induced hyperalgesia	[45]
			Hemorrhage in the ventral diencephalon, mesencephalon, and Basal ganglia, and severe white matter edema in the cerebral hemispheres and pontine nuclei and medullary tegmental	*Cognitive* impairment, necrosis, and death	[46, 47]
